# Dynamics of non-structural carbohydrates in three Mediterranean woody species following long-term experimental drought

**DOI:** 10.3389/fpls.2013.00400

**Published:** 2013-10-11

**Authors:** Teresa Rosas, Lucía Galiano, Romà Ogaya, Josep Peñuelas, Jordi Martínez-Vilalta

**Affiliations:** ^1^Centre de Recerca Ecològica i Aplicacions Forestals (CREAF)Cerdanyola del Vallès, Spain; ^2^Departament de Biologia Animal, Biologia Vegetal i Ecologia, Universitat Autònoma de BarcelonaCerdanyola del Vallès, Spain; ^3^Leibniz Centre for Agricultural Landscape Research (ZALF), Institute for Landscape BiogeochemistryMüncheberg, Germany; ^4^Consejo Superior de Investigaciones Científicas, Global Ecology Unit CREAF-CEAB-CSIC-UABCerdanyola del Vallès, Spain

**Keywords:** drought, crown condition, growth, long-term, non-structural carbohydrates, starch, throughfall manipulation

## Abstract

Stored non-structural carbohydrates (NSC) have been proposed as a key determinant of drought resistance in plants. However, the evidence for this role is controversial, as it comes mostly from observational, short-term studies. Here, we take advantage of a long-term experimental throughfall reduction to elucidate the response of NSC to increased drought 14 years after the beginning of the treatment in three Mediterranean resprouter trees (*Quercus ilex* L., *Arbutus unedo* L. and *Phillyrea latifolia* L.). In addition, we selected 20 *Q. ilex* individuals outside the experimental plots to directly assess the relationship between defoliation and NSC at the individual level. We measured the seasonal course of NSC concentrations in leaves, branches and lignotuber in late winter, late spring, summer, and autumn 2012. Total concentrations of NSC were highest in the lignotuber for all species. In the long-term drought experiment we found significant depletion in concentrations of total NSC in treatment plots only in the lignotuber of *A. unedo*. At the same time, *A. unedo* was the only species showing a significant reduction in BAI under the drought treatment during the 14 years of the experiment. By contrast, *Q. ilex* just reduced stem growth only during the first 4 years of treatment and *P. latifolia* remained unaffected over the whole study period. However, we found a clear association between the concentrations of NSC and defoliation in *Q. ilex* individuals sampled outside the experimental plots, with lower total concentrations of NSC and lower proportion of starch in defoliated individuals. Taken together, our results suggest that stabilizing processes, probably at the stand level, may have been operating in the long-term to mitigate any impact of drought on NSC levels, and highlight the necessity to incorporate long-term experimental studies of plant responses to drought.

## Introduction

Climate-related tree mortality has been observed worldwide in all major forest biomes (Allen et al., 2010). Severe and recurrent droughts have been identified as a major contributing factor of forest decline and mortality in central Europe (Bréda et al., 2006; Rigling et al., 2013) and in the Mediterranean basin (e.g., Peñuelas et al., 2001; Martínez-Vilalta et al., 2002; Galiano et al., 2010). Because more frequent and intense droughts are predicted, particularly in the Mediterranean basin (IPCC, 2007), different sensitivity of species to drought may cause widespread changes in species distribution and community composition (Engelbrecht et al., 2007; Choat et al., 2012; but see Lloret et al., 2012). Such vegetation shifts may have important implications for ecosystem function, land–atmosphere interactions and ecosystem services to humans in general (Dale et al., 2000; Bonan, 2008; Anderegg et al., 2012a).

Despite a growing research interest on the physiological mechanisms underlying drought-induced mortality, these mechanisms are still poorly understood. McDowell et al. (2008) formalized two main hypotheses: hydraulic failure and carbon starvation. Hydraulic failure is hypothesized to occur when drought intensity is sufficient to cause generalized cavitation in xylem conduits as a result of very negative xylem water potentials, to the point that the water column is broken and the water transport system is impaired (Tyree and Sperry, 1989; Sparks and Black, 1999). The carbon starvation hypothesis predicts that stomatal closure to prevent desiccation causes photosynthetic carbon uptake to diminish to near zero, and that continued demand for carbohydrates to maintain metabolism will deplete carbohydrate reserves, leading eventually to starvation and death (McDowell et al., 2008; Adams et al., 2009). Biotic agents, such as insects and pathogens, can amplify or be amplified by both carbon starvation and hydraulic failure (Shaw et al., 2005; Fettig et al., 2007). However, these non-exclusive hypotheses are still controversial (Sala and Hoch, 2009; Sala et al., 2010, 2012; McDowell et al., 2011). Recent research has emphasized the links between the two previous hypotheses through the hydraulic system of plants (McDowell, 2011) and how tree mortality implies a complex cascade of changes involving interconnected plant systems over multiple timescales (Anderegg et al., 2012b).

Although carbon reserve depletion has been considered one of the most critical aspects for tree survival under drought (McDowell et al., 2011; Sala et al., 2012), the role of stored non-structural carbohydrates (NSC) in tree's ability to cope with stress is not well established. For instance, Adams et al. (2009) investigated the response of trees to drought and increased temperature under controlled environmental conditions and attributed the earlier death of the trees under warmer temperatures to increased respiration and consequent depletion of carbon reserves, although hydraulic failure also occurred at death. However, the study was criticized for its methodology (Leuzinger et al., 2009) and for not adequately addressing alternative hypothesis, for instance that drought could impact the mobilization and long-distance transport of stored reserves even if the stored C pool wasn't entirely consumed (Sala, 2009; Sala et al., 2010). Recently, Adams et al. (2013) provided additional evidence supporting and refining the previous conclusion of a role of carbon metabolism in the mechanism of drought-induced mortality, at least for piñon pine. There is a growing number of studies showing an association between carbon reserves depletion and drought induced mortality in different species (Piper, 2011; Galiano et al., 2011; Adams et al., 2013; Galvez et al., 2013; Hartmann et al., 2013; Mitchell et al., 2013), but there are also counterexamples [e.g., Anderegg et al. (2012c); Gruber et al. (2012); Mitchell et al. (2013) for two of the three species they studied]. Overall, most studies have found either no reduction or even increases in carbohydrate reserves under moderate drought (Sala and Hoch, 2009; Galvez et al., 2011; Woodruff and Meinzer, 2011; Anderegg, 2012; Anderegg et al., 2012c), which is consistent with the fact that growth is considered to be more sensitive to drought than assimilation (cf. Sala et al., 2010). Modeling results and some experimental evidence (cf. above) suggest, however, that carbohydrate reserves should decline under exceptionally long or severe droughts (McDowell et al., 2011). Recent research has shown that mortality mechanisms may not be defined at the organism level but rather within tree compartments (Hartmann et al., 2013), thus measurements on different organs will be necessary to understand physiological responses to drought.

Carbohydrate storage has been traditionally considered a measure of carbon shortage or surplus for growth that reflects the tree carbon source-sink balance: storage increases when source supply by photosynthesis exceeds demands for growth and maintenance, and decreases when demands exceeds supply (Mooney, 1972; Chapin et al., 1990; Körner, 2003). This reasoning also assumes that NSC storage could be used by trees to cope with stress and, under carbon deficit conditions, stored carbon would be used until reserves are exhausted (Adams et al., 2009; McDowell et al., 2011). However, an alternative view has been recently proposed considering the NSC pool as an active sink that competes with growth under water stress conditions, suggesting that trees actively regulate storage at the expense of short-term growth to optimize growth and survival in the long term (Sala et al., 2012; Wiley and Helliker, 2012). Studies examining NSC in trees under varying conditions of water availability support that drought-related reductions in growth are not likely to be caused by constraints on carbon availability (Körner, 2006; Millard et al., 2007; Sala and Hoch, 2009; Woodruff and Meinzer, 2011). Another critical (and unexplored) aspect is that a significant fraction of C allocated to the NSC pool could be sequestered, becoming unavailable for the tree, which could lead to temporary deficits in carbon supply, eventually reducing tree vigour and potentially leading to tree mortality (Hoch et al., 2003; Millard et al., 2007; Gruber et al., 2012).

If the short-term responses of stored NSC reserves to drought remain unclear, much less is known about their response to chronic drought. This is particularly critical for the understanding and prediction of tree and forest responses to future climate scenarios (IPCC, 2007). Mediterranean-type ecosystems have been traditionally considered highly resilient to disturbances (Westman, 1986). A key element of resilience is that many Mediterranean shrubs and trees have efficient regeneration strategies (e.g., resprouting) that allow for rapid recovery after disturbances (Lavorel, 1999). However, resprouting generally depends on storage organs such as lignotubers (Canadell and López-Soria, 1998) that can be depleted during tree resprouting after disturbance (e.g., experimental logging, Canadell and López-Soria, 1998; severe drought, Galiano et al., 2012). Recurrent droughts may force individuals to use up carbon reserves to resprout and recover, and would produce a progressive loss of resilience by depleting the ability to regenerate (López et al., 2009; Lloret et al., 2004). At the same time, however, there is a plethora of stabilizing processes, acting at scales ranging from the individual to the whole community, which may provide effective mechanisms to mitigate the effects of drought events and maximize resilience (Lloret et al., 2012).

This study takes advantage of a long-term drought simulation experiment that has been running since 1999 in a Mediterranean forest. This experiment has provided evidence of different drought sensitivity across the three dominant woody species, with *Phillyrea latifolia* L. being less affected by increased drought than *Quercus ilex* L. and *Arbutus unedo* L. (Martínez-Vilalta et al., 2003; Ogaya et al., 2003; Ogaya and Peñuelas, 2007a,b; Barbeta et al., 2013). However, the effect of the drought treatment on stem growth and mortality in *A. unedo* and *Q. ilex* was attenuated as the study progressed (Barbeta et al., 2013).

Here, we measured the concentrations of stored NSCs in leaves, branches and lignotubers of these three co-occurring evergreen species growing in control and droughted plots. We aimed at (1) characterizing the seasonal change in NSC storage over the course of one year in three evergreen Mediterranean species, as well as the relationship between NSC concentrations and growth; (2) determining the effect of long-term drought in the amount and composition of NSC in the previous species; and (3) assessing the relationship between canopy condition and NSC concentrations in *Q. ilex* individuals, as this species has suffered recent events of drought-induced defoliation in the study area.

## Materials and methods

### Study site and experimental design

**Figure 1:**
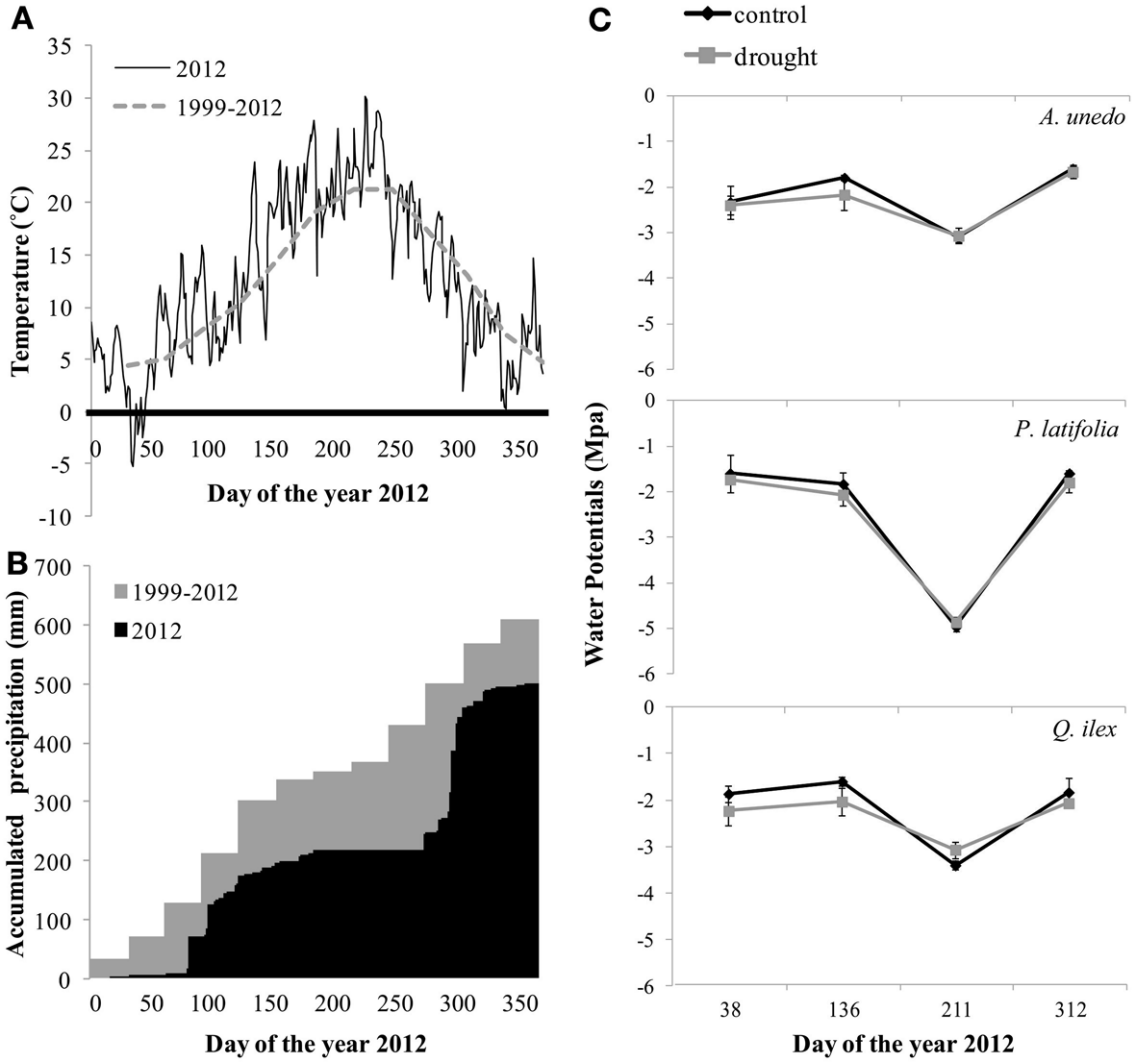
**Environmental variables during the period of study. (A)** Daily temperature in 2012 and monthly average temperature during the years 1999–2012, **(B)** daily accumulated precipitation in 2012 and monthly accumulated precipitation during the years 1999–2012 and **(C)** seasonal patterns of midday leaf water potentials during 2012. Bars represent standard error (*n* = 3 plot averages).

The study was carried out in a holm oak (*Quercus ilex* L.) forest in the Prades Mountains, NE Spain, located on the south-facing upper slopes of the Torners valley (41°21′ N, 1°2′ E; 990 m asl). The climate is Mediterranean, with a mean annual rainfall of 609 mm and a mean annual temperature of 12.2°C (climate data for the period 1999–2012 from an automatic meteorological station installed at the site) (Figures 1A,B). Summer drought is pronounced, with 65.3 mm of rain on average from mid-June to mid-September. The soil is a Dystric Cambisol over Paleozoic schist, ranging between 35 and 90 cm in depth. Bedrock outcrops are frequent and the terrain slope is around 25%. The holm oak forest is dominated by *Q. ilex* L., *P. latifolia* L. and *A. unedo* L. There are other evergreen species well adapted to dry conditions (*Erica arborea* L., *Juniperus oxycedrus* L., *Cistus albidus* L.) and occasional individuals of deciduous species (*Sorbus torminalis* (L.) Crantz, *Acer monspessulanum* L.)

**Figure A1:**
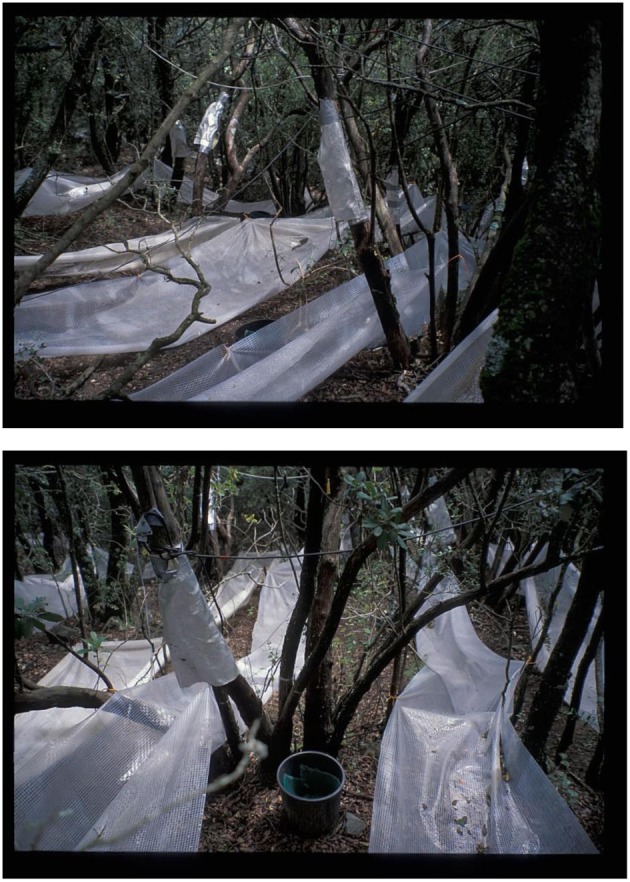
**Photographs of the drought experimental plots**.

A long-term drought simulation experiment has been running in the study area since 1999 (Martínez-Vilalta et al., 2003; Ogaya et al., 2003; Ogaya and Peñuelas, 2007a,b; Barbeta et al., 2013). The experimental design includes eight 15 × 10 m plots located at the same altitude along the slope. Half of the plots (randomly selected) received the drought treatment and the other half did not receive any treatment and were considered control plots. The drought treatment consisted of partial rainfall exclusion achieved by suspending 14 m long by 1 m wide transparent PVC strips to channel water outside the plots, at a height of 0.5–0.8 m above the soil (see Appendix Figure A1). Strips covered approximately 30% of the total plot surface. In addition, a 90 cm deep ditch was excavated along the entire top edge of the upper part of the treatment plots to intercept runoff water. Litter falling on the plastic strips was regularly placed below them to ensure that differences in the contents of soil nutrients among treatment and control plots were attributable only to the availability of water for the decomposition of this litter (Ogaya and Peñuelas, 2007b).

Soil water content and leaf water potentials were monitored during the 14 years of drought simulation experiment. Fully exposed leaves were collected from the upper part of the canopy of five individuals per species and plot. Leaf water potentials were measured immediately after leaf collection at midday (1100–1300 h, solar time) using a Scholander-type chamber. Measurements were conducted once per season over the whole 14 years period. Over the overall studied period (1999–2012), soil water content was significantly lower in the drought treatment compared with the control plots (−18.06% ± 3.02); the effect ranging from 25–30% in volume during the rainy seasons to 5–10% during summer droughts (Barbeta et al., 2013). Annual measurements of the diameter (at 50 cm height) of all living stems larger than 2 cm allowed us to estimate basal area increment (BAI) for the whole studied period at both the individual and plot levels.

### Field sampling methods

In mid-April (late winter, before bud-break), mid-June (late spring), mid-August (summer) and late-October (autumn) 2012, 54 individuals were sampled from six of the experimental plots (Table 1) described in the previous section (three treatment and three control plots). The other two plots were not sampled because they did not contain individuals of the three study species. In each plot, we randomly selected three individuals of each species: *Q. ilex*, *P. latifolia*, and *A. unedo*. These same nine individuals per species were sampled over the study period.

**Table 1:** **Characterization of the six plots studied (mean ± *SE*, *n* = 3 plots)**.

		**Treatment plots**	**Control plots**
*Arbutus unedo* L.	Density 2013 (stems ha^−1^)	1022 ± 235	1822 ± 289
	Basal area 2013 (m^2^ ha^−1^)	4.418 ± 1.598	9.849 ± 2.520
	BAI 1999–2012 (m^2^ ha ^−1^ year^−1^)	0.014 ± 0.042	0.152 ± 0.027
	Annual stem mortality rate (1999–2012)	0.010 ± 0.008	0.006 ± 0.005
*Phillyrea latifolia* L.	Density 2013 (stems ha^−1^)	6600 ± 3936	5711 ± 3004
	Basal area 2013 (m^2^ ha^−1^)	7.950 ± 5.067	6.107 ± 3.650
	BAI 1999–2012 (m^2^ ha ^−1^ year^−1^)	0.057 ± 0.053	0.052 ± 0.028
	Annual stem mortality rate (1999–2012)	0.008 ± 0.005	0.006 ± 0.001
*Quercus ilex* L.	Density 2013 (stems ha^−1^)	4333 ± 1674	5644 ± 1325
	Basal area 2013 (m^2^ ha^−1^)	27.660 ± 12.497	34.192 ± 7.799
	BAI 1999–2012 (m^2^ ha^−1^ year^−1^)	0.001 ± 0.130	0.147 ± 0.135
	Annual stem mortality rate (1999–2012)	0.037 ± 0.017	0.021 ± 0.002
All species	Total basal area 1999 (m^2^ ha^−1^)	39.02 ± 8.08	45.24 ± 1.53
	Total basal area 2013 (m^2^ ha^−1^)	40.03 ± 8.83	50.15 ± 2.06

*Mean annual stem mortality rate was calculated as m = 1 − [1 − (No − Nt) / No]^1/t^ as described in Sheil et al. (1995), where No and Nt are the live stem counts at the beginning and the end of the corresponding time period (t = 14 years). Abbreviations: BAI, basal area increment*.

*Quercus ilex* individuals inside the plots did not show contrasted responses in terms of defoliation at the time of sampling. Thus, we selected 20 additional *Q. ilex* individuals outside the plots with different crown condition: 10 individuals with >50% of green leaves (healthy) (average ± standard error = 87.5% ± 2.0) and 10 individuals with <50% of green leaves (defoliated) (27.5% ± 3.7). We visually estimated the percentage of green leaves relative to the amount in a healthy canopy of a similar sized tree in the study area. This estimation was always carried out by the same observer to minimize error. The diameter at breast height (DBH) was measured for all living stems larger than 2 cm of each sampled individual.

In all cases, three different tissues (leaves, branches, and lignotuber) were collected from each sampled individual to measure the concentrations of stored NSCs. One-year old and current year leaves were collected for the first (late winter) and the other (late spring, summer, autumn) samplings, respectively. Most *P. latifolia* and *A. unedo* individuals had lost the one-year old leaves by the second sampling, hence, we decided to sample current year leaves for the three species in order to make the rest of measures of NSC comparable across species. To minimize diurnal variability in NSC, leaves were always collected between 12:00 pm and 3:00 pm (solar time). During the second sampling (late spring), an infestation by the caterpillar *Catocala nymphagoa* was detected in the study area, which mainly affected *Q. ilex* individuals. However, its effects did not differ between treatment and control plots (Ogaya et al., submitted). Branch samples were ~0.5 to 1 cm in diameter (with bark removed). Lignotuber samples were ~1 cm thick in all cases (with bark removed). Assays with lugol solution indicated that in *Q. ilex* most NSC are stored in this ~1 cm thick outer layer. In *P. latifolia* and in *A. unedo*, NSC may reach deeper lignotuber layers, up to 2 cm or more (data not shown). However, we decided to standardize samples at ca. 1 cm thick to allow direct comparability at the tissue level. Immediately following collection, all samples were stored inside paper bags and placed on ice in a cooler.

### Non-structural carbohydrates analyses

All samples were microwaved for 90 s within a few hours of collection to minimize continued enzymatic activity, oven-dried for 72 h at 65°C and ground to fine powder. Non-structural carbohydrates (NSC) were defined as free sugars (glucose and fructose), sucrose plus starch and were analyzed following the procedures described by Hoch et al. (2002) with some minor modifications (cf. Galiano et al., 2011). Sapwood powder (~12 to 14 mg) was extracted with 1.6 ml distilled water at 100°C for 60 min. After centrifugation, an aliquot of the extract was used for the determination of soluble sugars (glucose, fructose and sucrose), after enzymatic conversion of sucrose and fructose into glucose (invertase from *Saccharomyces cerevisiae* and glucose hexokinase (GHK) assay reagent, I4504 and G3293, Sigma-Aldrich, Spain). Another aliquot was incubated with an amyloglucosidase from *Aspergillus niger* (10115 Sigma-Aldrich) at 50°C overnight, to break down all NSC (starch included) to glucose. The concentration of free glucose was determined photometrically in a 96-well microplate reader (Sunrise™ Basic Tecan, Männedorf, Switzerland) after enzymatic (GHK assay reagent) conversion of glucose to gluconate-6-phosphate. The dehydrogenation of glucose causes an increase in optical density at 340 nm. Starch was calculated as total NSC minus soluble sugars. All NSC values are expressed as percent dry matter.

### Statistical analyses

We used general linear mixed models to study the seasonal patterns of midday leaf water potentials during 2012. We included species, treatment and season as explanatory fixed factors and midday leaf water potentials as a response variable. Plot effects were modeled as a random factor to account for the spatial autocorrelation among individuals within a plot.

Similar general linear mixed models were used to study the growth of the three co-occurring species (*Q. ilex*, *P. latifolia* and *A. unedo*) in terms of stem BAI in the two non-overlapping periods: 1999–2002 and 2003–2013. These periods separate an initial phase in which treatment effects on radial growth were observed for *A. unedo* and *Q. ilex* (Ogaya and Peñuelas, 2007b), from the following time period. In this case we included species, treatment and period as explanatory fixed factors and annual stem BAI of the corresponding period as response variable. Random effects included individual nested into plot, to account for temporal and spatial autocorrelation.

We also used general linear mixed models to assess the effect of individual- and plot-level variables on the amount of stored NSC. It should be noted that throughout the manuscript NSC is used to refer generically to NSC, while TNSC is used to refer specifically to the total value (sum of starch and soluble sugars).Two models were fitted for each species, one to study the total concentration of NSC (TNSC) and a second one to study its composition (soluble sugars vs. starch), expressed as the ratio of soluble sugars (glucose, fructose and sucrose) to TNSC. These two variables, TNSC and Soluble sugars:TNSC, were normally distributed and, thus, were directly used as the response variable in the first and the second model, respectively. In all cases we started with the same saturated model, including treatment (plot-level), tissue and season as explanatory fixed factors, and basal area and BAI as covariates, together with all two-level interactions. Random effects included individual nested into plot, to account for temporal and spatial autocorrelation. It should be noted that some *P. latifolia* individuals had zero NSC reserves and the ratio between soluble sugars and TNSC could not be calculated. These individuals were therefore not considered in the Soluble sugars:TNSC model. For all explanatory factors, one group was considered the reference, the effect for which was incorporated in the model intercept term, and the effect of each was considered relative to the reference. The reference tissue was lignotuber, the reference period was late winter, and the reference treatment was control.

Similarly, to assess the relationship between defoliation and NSC in *Q. ilex* individuals sampled outside the plots, we conducted two general linear mixed models with individual effects modeled as a random factor. As before, one model had the concentration of TNSC as response variable, whereas the other had the ratio between soluble sugars and TNSC. Explanatory fixed variables included crown condition, tissue and season as factors and basal area as a covariate. In this case, the reference tissue was lignotuber, the reference period was late winter, and the reference treatment was healthy individuals.

Finally, four general linear models were performed to investigate the relationship between growth and TNSC concentrations in the three co-occurring species: two with the seasonal variation of TNSC (difference between the maximum and minimum NSC value), in branches or lignotuber, as response variable; and the other two with the seasonal average of TNSC, again in branches or lignotuber, as response variable. Response variables were normally distributed in all cases. The four models had species identity as explanatory fixed factor and individual BAI of the last three years as covariate. *A. unedo* was considered the reference. We did not fit analogous models for TNSC in leaves because different cohorts were sampled in different sampling campaigns (cf. above) and, therefore, TNSC concentrations were not strictly comparable.

In all cases model selection was based on Akaike's information criterion (AIC). We started from the saturated model (with all two-order interactions) and progressively removed non-significant explanatory variables until a minimal adequate model with the lowest AIC was obtained. Models within 4 AIC units were considered equivalent and the simplest one was selected. Only in one case the difference between the simplest, selected model and the model with lowest AIC was higher than 2 AIC units (2 < ΔAIC < 4) and, in all cases, our main conclusions remain the same regardless of the specific model selection criteria we used (see Appendix Table A3). In all cases, the residuals of the selected models showed no obvious pattern and were approximately normally distributed. Statistical analyses were carried out with R version 2.13.2 (R Development Core Team, Vienna, Austria).

## Results

### Long-term drought experiment

**Figure 2:**
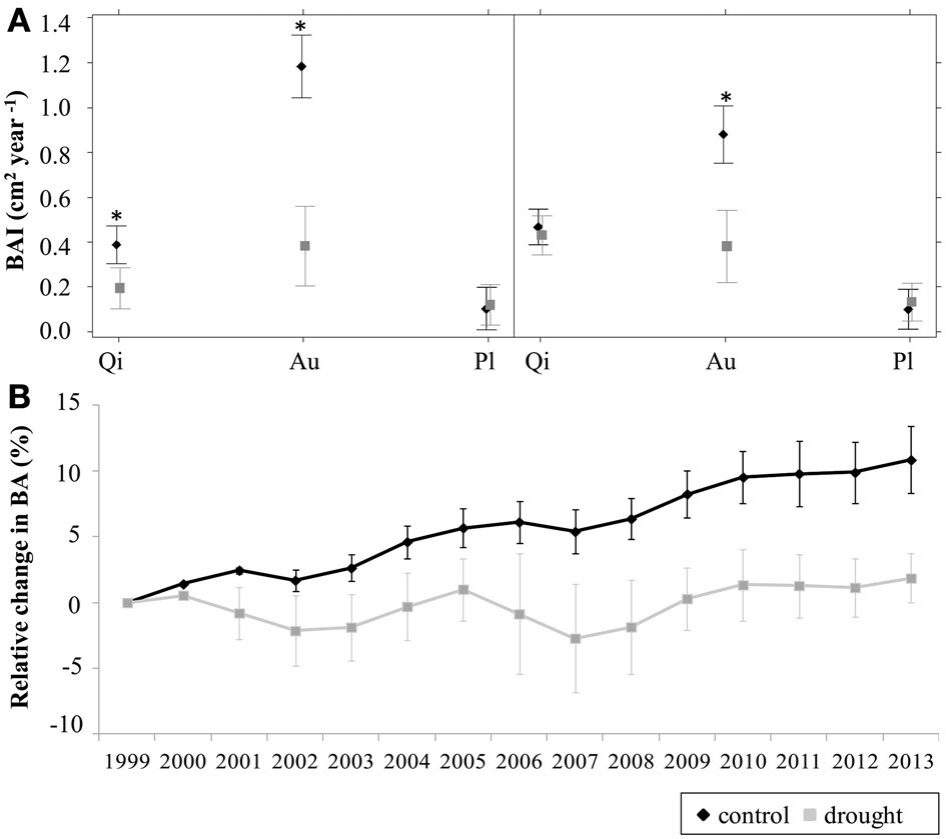
**(A)** Mean annual basal area increment (BAI) during the periods 1999–2002 and 2003–2012 for living stems of *Quercus ilex* (Qi), *Arbutus unedo* (Au) and *Phillyrea latifolia* (Pl) individuals in control (black) and drought (gray) plots, according to the results of the general linear mixed model used to fit BAI data (see text for details). Each symbol represents the value of the corresponding model coefficient, and bars represent their standard errors (at the plot level). Asterisks indicate significant differences between treatments within a species (*P* < 0.05). **(B)** Relative change in basal area (BA) during the overall studied period (cumulative from 1999) for living stems of all species in control (black) and drought (gray) plots. Bars represent standard errors (*n* = 3 plots).

Annual BAI differed across species in the two studied periods (1999–2002 and 2003–2013). *Q. ilex* showed lower BAI rates than *A. unedo* but higher than *P. latifolia* [Figure 2A; (see Appendix Table A2)]. *A. unedo* was the only species showing a significant reduction in BAI under the drought treatment in the two studied periods [Figure 2A; (see Appendix Table A2)]. *Q. ilex* only showed a reduction in droughted plots in the first period [Figure 2A; (see Appendix Table A2)] while *P. latifolia* did not show significant differences between treatments in any of the two periods. Using longer initial periods (e.g., 1999–2003) resulted in similar qualitative patterns, but the treatment effects on *Q. ilex* growth became non-significant, indicating that for this species treatment effects had largely disappeared by the fifth year of treatment (results not shown). At the plot level, the BAI dynamics of the dominant species resulted in a sustained increase in basal area in control plots from 1999 to 2013 (11% overall increase) (Figure 2B). In contrast, in drought plots basal area did not show any clear trend over the whole study period and there were two multi-year episodes of declining basal area (2001–2003 and 2006–2008) (Figure 2B).

The year 2012 was slightly warmer (Figure 1A) and clearly drier than the average of the 14 years of drought treatment, with a total annual rainfall of 500.7 mm, of which only 21.5 mm fell during the summer (mid-June to mid-September) (Figure 1B). However, there were no significant differences between treatment and control plots in midday leaf water potentials in 2012 in any of the studied species, suggesting that water availability per capita was similar regardless of the treatment [Figure 1C; (see Appendix, Table A1)].

**Figure 3:**
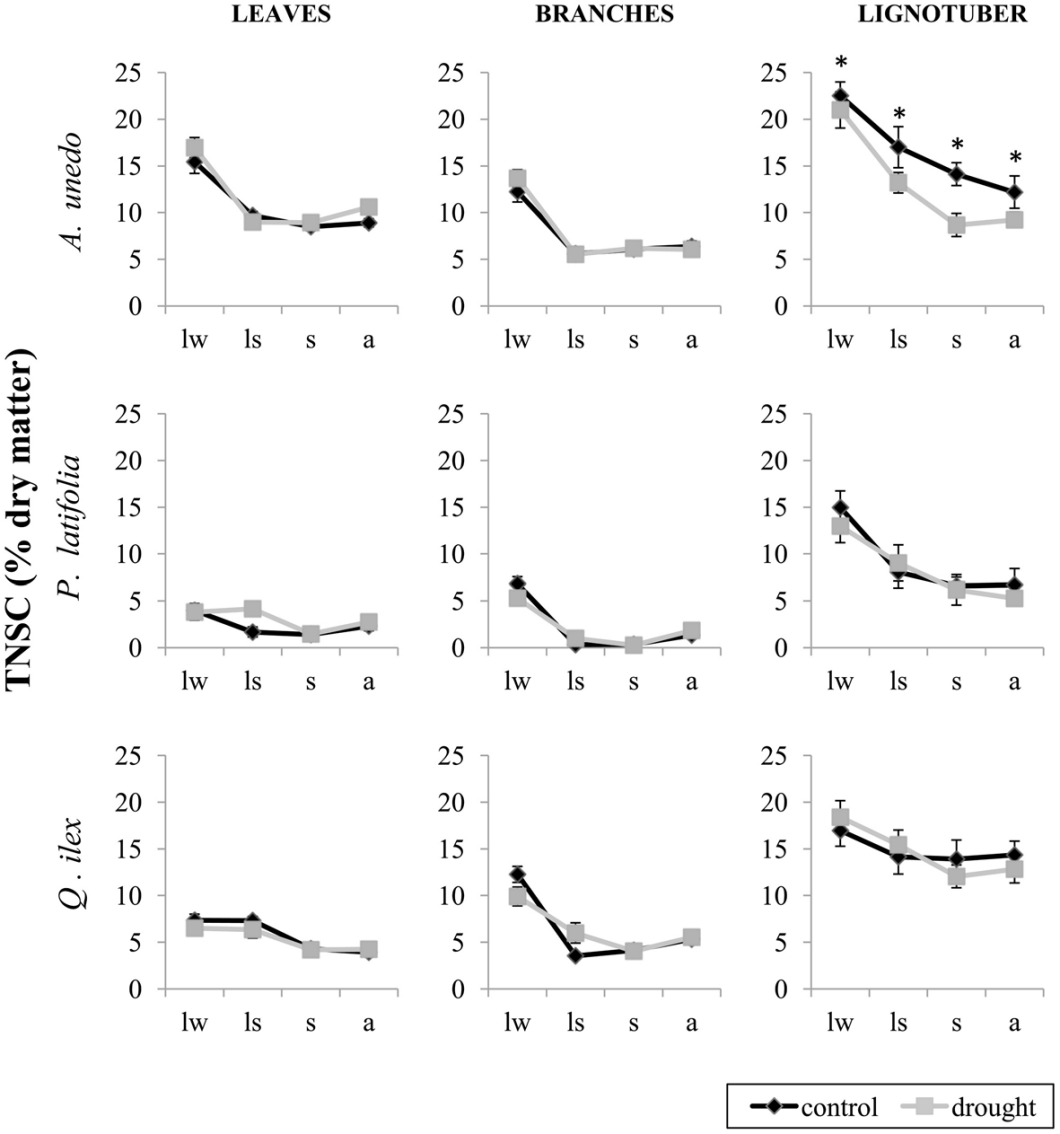
**Percent dry matter concentrations of total non-structural carbohydrates (TNSC) in leaves, branches and lignotuber of *Arbutus unedo, Phillyrea latifolia* and *Quercus ilex* in control (black) and drought (gray) plots**. Samples were collected in late winter (lw), in late spring (ls), in summer (s) and in autumn (a) 2012. Asterisks indicate significant differences between treatments (*P* < 0.05). Bars represent standard errors (*n* = 9 individuals).

TNSC concentrations tended to be highest in *A. unedo* and lowest in *P. latifolia* (Figure 3). Among tissues, TNSC concentrations were highest in the lignotuber for all species (Table 2A). The long-term drought experiment did not result in a general depletion of NSC reserves over the course of the studied year. We only found significant differences in the concentrations of TNSC between treatments in the lignotuber of *A. unedo*, with individuals in the droughted plots showing lower concentrations of TNSC (Table 2A). Some individuals of *P. latifolia* in late winter, late spring and summer reduced NSC to zero in both control and treatment plots.

**Table 2:** **Summary of the models for (A) total non-structural carbohydrates (TNSC) and (B) soluble sugars fraction for the individuals sampled inside the experimental plots (see text)**.

	***Arbutus unedo* L**.					***Phillyrea latifolia* L**.			***Quercus ilex* L**.			
**Fixed effects**	**Estimate**	***SE***	***DF***	***P*-value**	**Estimate**	***SE***	***DF***	***P*-value**	**Estimate**	***SE***	***DF***	***P*-value**
**(A)**												
(Intercept)	23.470	0.859	185	ns	14.571	0.834	184	^***^	17.672	0.757	187	^***^
Drought	−3.434	0.846	4	^*^	−1.233	0.889	4	ns	–	–	–	–
Autumn	−11.047	1.007	185	^***^	−8.514	1.096	184	^***^	−4.091	0.985	187	^***^
Late spring	−6.659	1.007	185	^***^	−6.724	1.096	184	^***^	−2.886	0.985	187	^**^
Summer	−10.361	1.007	185	^***^	−8.115	1.096	184	^***^	−4.705	0.985	187	^***^
Branches	−10.667	1.126	185	^***^	−7.912	0.949	184	^***^	−6.600	0.985	187	^***^
Leaves	−7.651	1.126	185	^***^	−10.075	0.949	184	^***^	−10.756	0.985	187	^***^
Autumn:Branches	4.320	1.424	185	^**^	3.526	1.342	184	^**^	−1.550	1.393	187	ns
Late spring:Branches	−0.733	1.424	185	ns	0.033	1.342	184	ns	−3.430	1.393	187	^*^
Summer:Branches	3.546	1.424	185	^*^	1.832	1.342	184	ns	−2.286	1.393	187	ns
Autumn:leaves	4.595	1.424	185	^**^	6.625	1.342	184	^***^	1.246	1.393	187	ns
Late spring:leaves	−0.196	1.424	185	ns	4.454	1.342	184	^**^	2.808	1.393	187	^*^
Summer:leaves	2.869	1.424	185	^*^	5.125	1.342	184	^***^	2.019	1.393	187	ns
Drought:branches	3.721	1.007	185	^***^	–	–	–	–	–	–	–	–
Drought:leaves	4.184	1.007	185	^***^	–	–	–	–	–	–	–	–
Drought:autumn	–	–	–	–	1.071	1.096	184	ns	–	–	–	–
Drought:late spring	–	–	–	–	2.612	1.096	184	^*^	–	–	–	–
Drought:summer	–	–	–	–	1.070	1.096	184	ns	–	–	–	–
**(B)**												
(Intercept)	0.302	0.036	187	^***^	0.025	0.048	173	ns	0.160	0.039	187	^***^
Autumn	0.520	0.048	187	^***^	0.216	0.062	173	^***^	0.339	0.050	187	^***^
Late spring	0.171	0.048	187	^***^	0.070	0.062	173	ns	0.057	0.050	187	ns
Summer	0.357	0.048	187	^***^	0.071	0.062	173	ns	0.296	0.050	187	^***^
Branches	0.339	0.048	187	^***^	0.015	0.062	173	ns	0.074	0.050	187	ns
Leaves	0.290	0.048	187	^***^	−0.026	0.064	173	ns	0.663	0.050	187	^***^
Autumn:branches	−0.182	0.067	187	^**^	0.019	0.088	173	ns	0.150	0.071	187	^*^
Lates spring:branches	0.128	0.067	187	ns	0.495	0.093	173	^***^	0.333	0.071	187	^***^
Summer:branches	−0.002	0.067	187	ns	0.133	0.090	173	ns	0.414	0.071	187	^***^
Autumn:leaves	−0.431	0.067	187	^***^	−0.169	0.089	173	ns	−0.330	0.071	187	^***^
Late spring:leaves	0.064	0.067	187	ns	0.176	0.091	173	ns	−0.280	0.071	187	^***^
Summer:leaves	−0.048	0.067	187	ns	−0.004	0.090	173	ns	−0.192	0.071	187	^**^

Different models were fitted for each species. The estimate value indicates the difference from the reference condition. In both cases the reference tissue was “lignotuber,” the reference period was “late winter,” and the reference treatment was “control.” Abbreviations:

* 0.01 < P < 0.05;

** 0.001 < P < 0.01;

*** P < 0.001;

ns, P > 0.05;

*–, not included in the best model*.

In *A. unedo*, TNSC concentrations decreased in all tissues in late spring and only in lignotuber tissue this reduction continued during the summer (Figure 3; Table 2A). TNSC concentrations in leaves were lower than in lignotuber, but higher than in branches (Table 2A). *P. latifolia* and *Q. ilex* individuals showed similar TNSC dynamics. In both species, TNSC concentrations changed significantly over seasons, and differed significantly between tissues. The effect of season on TNSC also differed significantly between tissues (season-tissue interaction). In woody tissues, TNSC values declined in late spring (Figure 3). Note, however, that sampled leaves belonged to different cohorts in late winter and the rest of sampling moments (see Materials and Methods). In *Q. ilex* the spring TNSC reduction was more pronounced in branches than in lignotuber (Table 2A). The seasonal reduction in the concentrations of TNSC was lower in treatment plots for *P. latifolia*, particularly between late winter and late spring (Table 2A).

**Figure 4:**
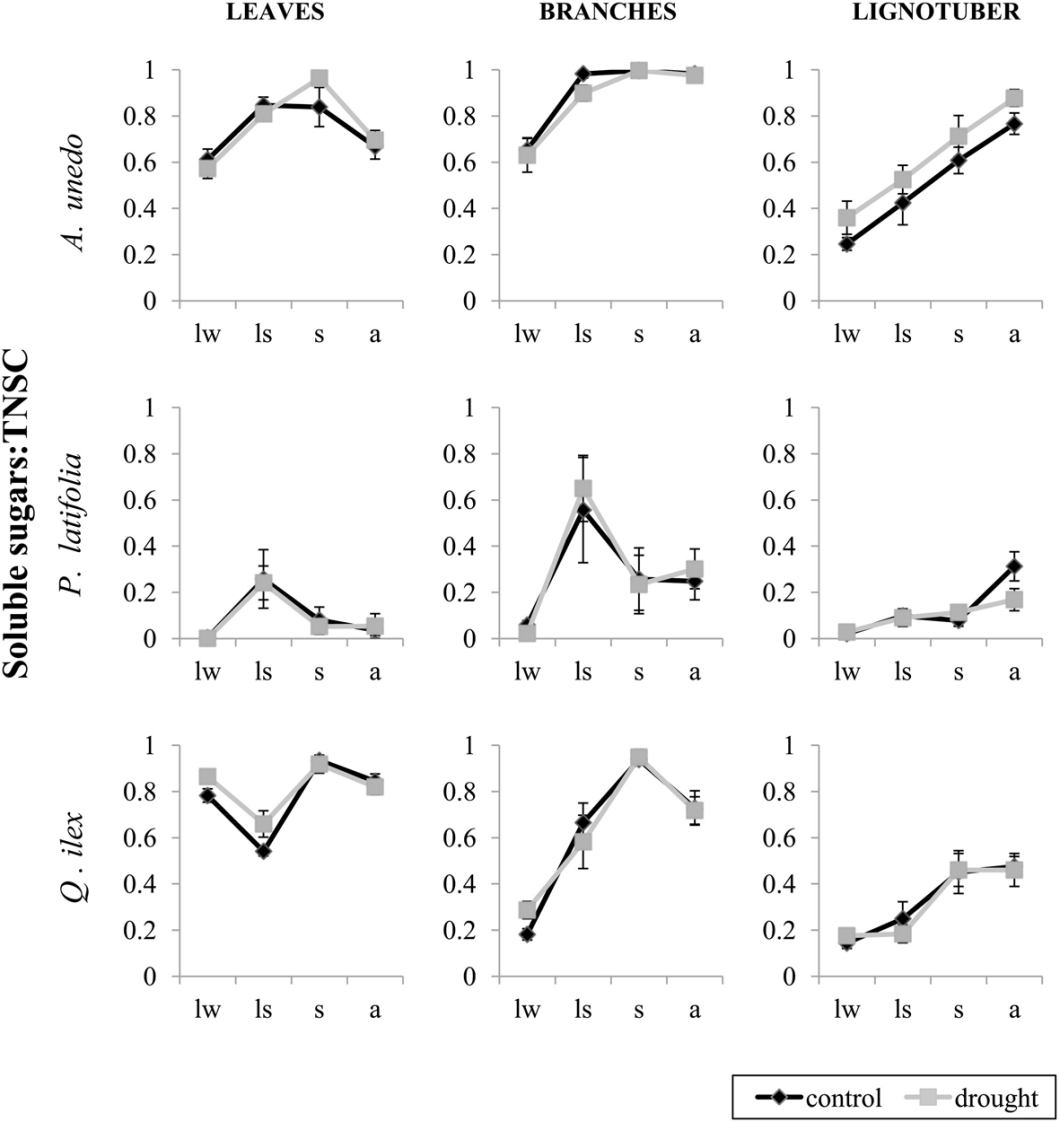
**Ratio between soluble sugars and total non-structural carbohydrates (TNSC) in leaves, branches and lignotuber of *Arbutus unedo, Phillyrea latifolia* and *Quercus ilex* in control (black) and drought (gray) plots**. Samples were collected in late winter (lw), in late spring (ls), in summer (s) and in autumn (a) 2012. Bars represent standard errors (*n* = 9 individuals).

The composition of NSC (soluble sugars vs. starch) changed significantly over seasons and between tissues. The effect of season on the composition of NSC also differed significantly between tissues (season-tissue interaction) in all species, but treatment effects were never significant. *P. latifolia* showed very low overall concentrations of soluble sugars with its proportion reaching the highest value in late spring in leaves and branches, whereas it increased significantly in autumn in the lignotuber (Figure 4; Table 2B). In *A. unedo*, there was also an increase in the proportion of soluble sugars in late spring. This increase was maintained until summer in leaves and over the rest of sampled seasons in branches, whereas the soluble sugars fraction continued increasing until autumn in the lignotuber (Figure 4; Table 2B). The soluble sugars fraction in lignotuber was significantly lower than in leaves and branches in all seasons except in autumn, when leaves had the lowest amount (Table 2B). In contrast to the other two species, the soluble sugars fraction of leaves in *Q. ilex* individuals decreased significantly in late spring and increased in summer. In branches, however, the proportion of soluble sugars increased significantly in late spring but reached the highest value in summer, while in lignotuber we only found a significant increase in summer (Figure 4; Table 2B).

We did not find any relationship between growth of the last three years and NSC concentrations in any species or tissue (Tables 3A,B). We performed the same models taking into consideration only the BAI of the last year (2012–2013), and we did not find any relationship either (results not shown). These models, however, confirmed the presence of significant differences in NSC concentrations between species. In branches, seasonal average TNSC concentrations were highest in *A. unedo* and lowest in *P. latifolia* (Table 3A). In lignotuber, *A. unedo* and *Q. ilex* values were not significantly different, but both were higher than those for *P. latifolia* (Table 3B). With regard to the seasonal variation of TNSC, we found significant differences between species for both tissues: *P. latifolia* had the lowest variation in branches (Table 3A) and *Q. ilex* in lignotuber (Table 3B).

**Table 3:** **Summary of the models of NSC as a function of growth (BAI 2010–2013) (A) in branches and (B) in lignotuber for individuals sampled inside the experimental plots (see text)**.

	**TNSC seasonal average**			**TNSC seasonal variation**		
**Fixed effects**	**Estimate**	***SE***	***P*-value**	**Estimate**	***SE***	***P*-value**
**(A)**						
Intercept	7.691	0.239	^***^	8.531	0.664	^***^
*P. latifolia*	−5.629	0.409	^***^	−3.104	1.135	^**^
*Q. ilex*	−1.314	0.333	^***^	−0.826	0.925	ns
BAI	0.009	0.026	ns	−0.067	0.071	ns
*P. latifolia*:BAI	0.175	0.524	ns	1.051	1.456	ns
*Q. ilex*:BAI	0.085	0.067	ns	0.267	0.185	ns
**(B)**						
Intercept	14.336	0.871	^***^	12.910	1.113	^***^
*P. latifolia*	−5.322	1.485	^***^	−2.425	1.902	ns
*Q. ilex*	0.453	1.217	ns	−4.144	1.551	^*^
BAI	0.159	0.098	ns	−0.107	0.120	ns
*P. latifolia*:BAI	−0.827	1.976	ns	0.345	2.441	ns
*Q. ilex*:BAI	−0.072	0.243	ns	0.181	0.310	ns

One single model was fitted in each case including the three species. The estimate value indicates the difference from the reference species “A. unedo.” Abbreviations:

* 0.01 < P <0.05;

** 0.001 < P < 0.01;

*** P < 0.001;

*ns, P > 0.05; BAI. BASAL area increment*.

### *Q. ilex* individuals with different crown condition

**Figure 5:**
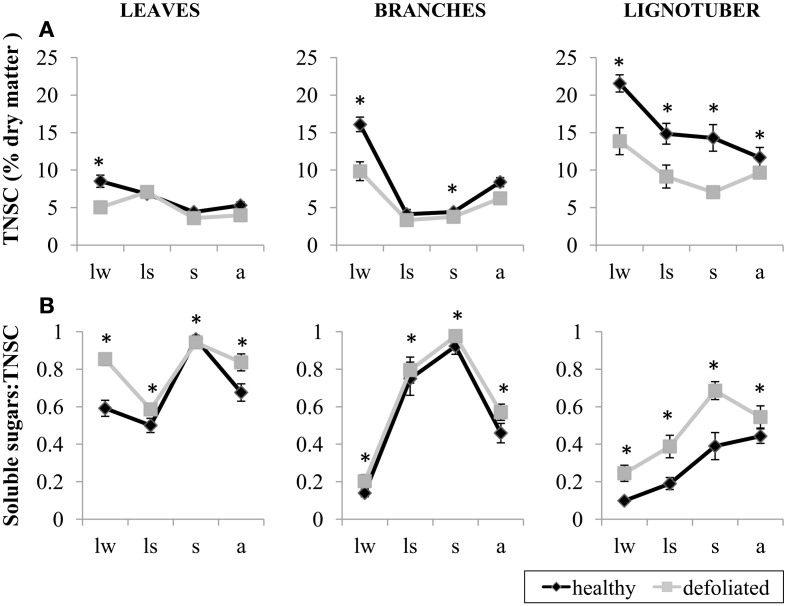
**(A)** Percent dry matter concentrations of total non-structural carbohydrates (TNSC) and **(B)** the ratio between soluble sugars and total non-structural carbohydrates (TNSC) in leaves, branches and lignotuber of *Quercus ilex* individuals sampled outside the experimental plots, as a function of defoliation: healthy canopy with >50% green leaves (black) and defoliated canopy with <50% green leaves (gray). Samples were collected in late winter (lw), in late spring (ls), in summer (s) and in autumn (a) 2012. Asterisks indicate significant differences between healthy and defoliated individuals (*P* < 0.05). Bars represent standard errors (*n* = 10 individuals).

Clear differences in the concentrations of TNSC were observed between *Q. ilex* individuals selected outside the plots with different levels of defoliation. TNSC concentrations were significantly affected by crown condition, tissue, season and their interactions (Table 4A). TNSC concentrations were higher in healthy individuals in all seasons in lignotuber, in late winter and in summer in branches, and only in late winter in leaves (Figure 5A; Table 4A). Lignotuber tissue reached the highest TNSC concentrations and woody tissues showed a significant TNSC reduction in late spring, more pronounced in branches than in lignotuber and in healthy than in defoliated individuals (Figure 5A; Table 4A). We found differences in soluble sugar fraction between healthy and defoliated individuals in all tissues, with healthy individuals showing lower concentrations (i.e., more starch; Figure 5B, Table 4B). Lignotuber was the tissue with the lowest soluble sugar fraction in late spring and summer. In late winter and in autumn no significant differences were observed between branches and lignotuber (Table 4B). Temporal patterns were similar to those previously described for *Q. ilex* individuals within experimental plots (Figure 5B). In leaves, there was a decrease of soluble sugars in late spring, followed by an increase in summer and decline to earlier winter levels in autumn (Figure 5B). In branches and lignotuber, the proportion of soluble sugars increased until summer, although this increase was stronger in branches (Figure 5B; Table 4B).

**Table 4:** **Summary of the models for (A) total non-structural carbohydrates (TNSC) and (B) soluble sugars fraction for *Q. ilex* individuals sampled outside the plots (see text)**.

**Fixed effects**	**Estimate**	***SE***	***DF***	***P*-value**
**(A)**				
(Intercept)	21.857	0.817	204	^***^
Defoliated	−8.314	0.958	18	^***^
Autumn	−9.019	1.054	204	^***^
Late spring	−7.577	1.054	204	^***^
Summer	−8.481	1.054	204	^***^
Branches	−6.337	1.021	204	^***^
Leaves	−13.095	1.021	204	^***^
Defoliated:autumn	3.968	1.054	204	^***^
Defoliated:late spring	3.722	1.054	204	^***^
Defoliated:summer	2.905	1.054	204	^**^
Defoliated:branches	3.201	0.913	204	^***^
Defoliated:leaves	4.316	0.913	204	^***^
Autumn:branches	1.379	1.291	204	ns
Late spring:branches	−3.525	1.291	204	^**^
Summer:branches	−1.836	1.291	204	ns
Autumn:leaves	4.906	1.291	204	^***^
Lates spring:leaves	5.899	1.291	204	^***^
Summer:leaves	4.280	1.291	204	^**^
**(B)**				
(Intercept)	0.109	0.035	209	^**^
Defoliated	0.126	0.020	18	^***^
Autumn	0.322	0.047	209	^***^
Late spring	0.117	0.047	209	^*^
Summer	0.366	0.047	209	^***^
Branches	−0.001	0.047	209	ns
Leaves	0.551	0.047	209	^***^
Autumn:branches	0.021	0.066	209	ns
Late spring:branches	0.484	0.066	209	^***^
Summer:branches	0.413	0.066	209	^***^
Autumn:leaves	−0.289	0.066	209	^***^
Lates spring:leaves	−0.297	0.066	209	^***^
Summer:leaves	−0.137	0.066	209	^*^

The estimate value indicates the difference from the reference condition. In both cases the reference tissue was “lignotuber,” the reference period was “late winter,” and the reference canopy state was “healthy.” Abbreviations:

* 0.01 < P < 0.05;

** 0.001 < P < 0.01;

*** P < 0.001;

*ns, P > 0.05*.

## Discussion

### Distribution and temporal variation of NSC concentrations across species

The NSC concentrations measured in the present study were comparable to the values previously reported for the same species [e.g., in *Q. ilex* lignotuber by López et al. (2009) and Galiano et al. (2012) and in *A. unedo* leaves by Meletiou-Christou et al. (1994)]. In our case, the concentrations of NSC and soluble sugars tended to be highest in *A. unedo* and lowest in *P. latifolia*. Meletiou-Christou et al. (1994) also found the highest amount of soluble sugars in *A. unedo* compared to other Mediterranenan evergreen sclerophylls.

It is normally assumed that resprouter species contain more TNSC than those plants that cannot resprout (Verdaguer and Ojeda, 2002), especially in their resprouting organs (Olano et al., 2006). In our case, the lignotuber was the tissue with the highest concentrations of TNSC and starch, in agreement with the function of lignotuber as a reservoir of dormant buds, carbohydrates and nutrients to ensure rapid regrowth after disturbances (Canadell and Zedler, 1995). Some previous reports, however, are not consistent with this view. Cruz and Moreno (2001), for instance, found that in the Mediterranean shrub *Erica australis* most TNSC were contained in the roots, followed by similar TNSC concentrations in both lignotuber and stem tissues. The authors suggested that lignotubers may not have been selected as principal sites for carbohydrate storage to ensure resprouting after disturbance. It should be noted, however, that differences in TNSC concentrations in the different tissues may not necessarily indicate differences in the total content of TNSC, which depends on the concentration but also on the total biomass of each organ.

Seasonal patterns of TNSC were influenced by phenological events. In all studied species and woody tissues, bud-break was associated to a decrease of TNSC concentrations, suggesting that woody tissues provide carbon to support shoot growth. Körner (2003) also found, in four Mediterranean species, a maximum NSC concentration in leaves and branch wood at the end of winter, and a significant reduction during spring. It is noteworthy that in *P. latifolia* and *Q. ilex* we did not find a significant spring reduction in the concentrations of TNSC in leaves, although this result should be interpreted with caution because we sampled two different leaf cohorts (see Materials and Methods). In evergreen species, old leaves are assumed to play a large role in supplying carbon and nutrients for newly developing shoots (Chapin et al., 1990), even though woody structures are also known to contribute to the NSC requirements for spring growth (Cherbuy et al., 2001; Palacio et al., 2007).

Regarding the composition of NSC (soluble sugars vs. starch), we observed different dynamics depending on the tissue. On the one hand, the starch fraction in branches of all species was highest before bud-break, but during the spring reduction of the concentration of TNSC the soluble sugars fraction increased, suggesting that starch was partially mobilized and converted into soluble sugars to support metabolism during the active spring period. The high starch concentrations prior to the bud-break have also been observed in other Mediterranean evergreen trees (Meletiou-Christou et al., 1994; Cruz and Moreno, 2001) in evergreen conifers in temperate forests (Hoch et al., 2003) and in *Pinus sylvestris* in the dry inner alpine valleys (Gruber et al., 2012). In the case of leaves, we detected an opposite trend between *Q. ilex* and the other two studied species: while in *P. latifolia* and *A. unedo* there was also an increase of the proportion of soluble sugars in late spring, in *Q. ilex* this proportion was significantly reduced, suggesting a lower capacity to mobilize reserves.

### Drought effects on NSC concentrations

We found contrasting patterns when comparing the effects of the drought treatment and the effects of crown condition on NSC concentrations. On the one hand, a clear association between lower concentrations of carbon reserves and drought-induced defoliation was observed in *Q. ilex* individuals sampled outside the plots. This result is consistent with previous studies on the same or similar species (Bréda et al., 2006; Galiano et al., 2012) and adds to the literature linking defoliation induced by extreme drought with low NSC concentrations (cf. Galiano et al., 2011; Piper, 2011; Adams et al., 2013; Galvez et al., 2013; Hartmann et al., 2013; Mitchell et al., 2013). Although our design does not allow to distinguish unambiguously whether this decline in NSC concentrations is due to a reduction in carbon uptake (due to reduced photosynthetic area or low stomatal conductance), to an increase in carbon sinks (higher use of stored carbon) or both, the fact that starch proportion was lower in defoliated individuals is consistent with an active mobilization of starch stores, which could eventually result in reserve depletion. Soluble sugars have been suggested to be important to maintain cell turgor and may also be critical to maintain vascular integrity under fluctuating environmental conditions (Volaire, 1995; Sala et al., 2012). It should be also noted that defoliated *Q. ilex* trees were able to produce new spring leaves with TNSC concentrations similar to healthy individuals. In any case, low levels of NSC have been associated with increased risk of drought induced mortality (McDowell et al., 2008; Galiano et al., 2011), and may compromise long term resilience to drought or other disturbances in resprouting species such as *Q. ilex* (Lloret et al., 2004; Galiano et al., 2012).

On the other hand, the long-term drought experiment did not result in a consistent depletion of NSC reserves over the course of the studied year. Some studies examining NSC concentrations in trees under varying conditions of water availability have provided evidence that NSC pools in trees are not easily depleted (Körner, 2003; Millard et al., 2007) and that (moderate) drought stress may lead to growth reductions before any effects are observed on carbohydrate levels (Millard et al., 2007; Sala and Hoch, 2009; McDowell, 2011; Woodruff and Meinzer, 2011; Anderegg et al., 2012c; Gruber et al., 2012). We only detected significant differences between treatments in the concentrations of lignotuber reserves in *A. unedo*. In this species, the lower concentrations of NSC in the lignotuber of droughted individuals was associated with lower growth rates compared to control trees. These results are suggestive of drought-induced carbon limitation, but they are not conclusive, as the drought treatment could have also affected biomass allocation to different organs. Further research should be aimed at scaling up these differences in NSC at the individual level, accounting for the different growth rates and biomass of the respective organs under different treatments.

Interestingly, during the first five years of the drought simulation experiment, drought plots experienced higher mortality rates in all species (Ogaya and Peñuelas, 2007b) but considering the whole 14 years treatment period, higher mortality rates in drought plots were only observed in *Q. ilex* (Table 1; Barbeta et al., 2013). During the first period (1999–2002) considered in this study, droughted individuals reduced stem diameter increment in *Q. ilex* and especially in *A. unedo*, whereas *P. latifolia* did not experienced any decrease. However, in the second period studied (2003–2012), the effects of the drought treatment on growth declined in *A. unedo* and disappeared in *Q. ilex*, while *P. latifolia* remained unaffected. The fact that treatment differences in mortality and growth have been attenuated 14 years after the beginning of the experiment suggests that stabilizing processes minimizing the effects of experimental drought have been operating at the stand level and have mitigated differences between treatments. In addition, the fact that *Q. ilex* individuals suffering drought-induced defoliation in the same study area showed lower NSC levels (Figure 5) suggests that differences in NSC between drought treatments might have been initially present but have faded away as the experimental treatment progressed. Although we do not have NSC data available during the first years of the experiment and we did not find a current trade-off between growth and NSC concentrations, the changes in mortality and growth during the 14 years of treatment could reflect a trade-off between short-term growth and survival in the long-term, as suggested by Sala et al. (2012) and Wiley and Helliker (2012), so that surviving individuals are those that managed to maintain NSC concentration above their survival threshold.

Alternatively, stabilization processes at the population and community levels could provide effective mechanisms to balance the effects of increased drought over the mid-term, as recently proposed by Lloret et al. (2012) and supported by the recent results by Barbeta et al. (2013) in the same study system. Changes in growth and mortality rates have resulted in a 11% increase in basal area in control plots between 1999 and 2013, compared to a mere 2% increase in droughted plots over the same period (Table 1; Figure 2B). Added to the slightly higher basal area in control plots at the beginning of the experiment, this difference has resulted in 25% higher basal area in control plots by 2013 (Table 1). This difference could result in a proportionally higher decline of resource availability in control plots and a proportionally higher availability of resources in droughted plots by the end of the experiment, including nutrients (Sardans and Peñuelas, 2007), light or even water (on a per capita basis), as shown by the similar midday leaf water potentials in 2012 between control and drought treatments (Figure 1C). Total basal area and total tree density have been negatively related to growth and positively related to mortality in many studies, suggesting that denser stands are indeed more susceptible to drought (Klos et al., 2009; Galiano et al., 2011, 2012; Vilà-Cabrera et al., 2011).

In conclusion, our results add to the evidence showing that defoliation induced by extreme drought events is associated with low NSC concentrations, potentially hindering the capacity of individuals to recover after recurrent droughts (cf. Galiano et al., 2012). At the same time, however, only the lignotuber NSC concentrations of one of the three studied species (*A. unedo*) was affected 14 years after the beginning of a long-term drought simulation experiment. We show that stabilizing processes have been operating to mitigate the demographic impacts observed during the first years of drought treatment, and argue that these same processes could have reduced any impact of drought on NSC levels at the beginning of the experiment. Our results highlight the necessity to incorporate long-term experimental studies to understand the variety of responses under environmental change, in agreement with previous reports stressing that predictions of vegetation change due to climate warming based on experimental manipulation will differ depending on the time frame of the study (eg., Hollister et al., 2005). The integration of drought responses at different spatial (from individuals to communities) and temporal scales remains a challenge to predict how Mediterranean forests will respond to ongoing climate change.

### Conflict of interest statement

The authors declare that the research was conducted in the absence of any commercial or financial relationships that could be construed as a potential conflict of interest.

## Figures and Tables

**Table A3:**
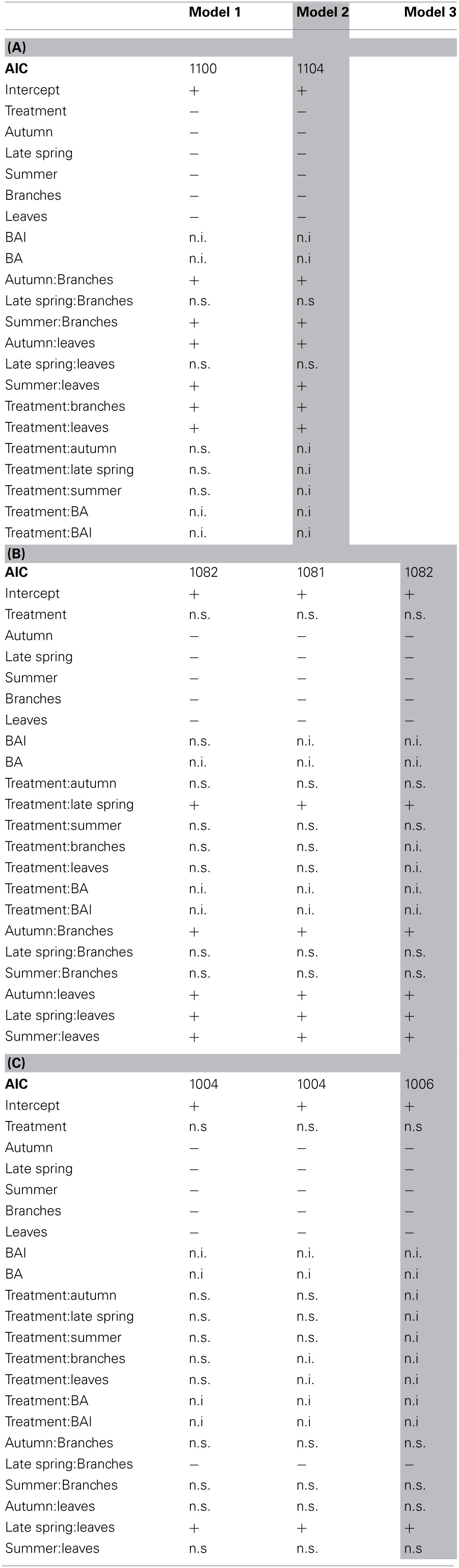
**Summary of the models for total non-structural carbohydrates within 4 AIC units of the model with lowest AIC for (A) *Arbutus unedo*, (B) *Phyllirea latifolia* and (C) *Quercus ilex***.

*The last model (shaded) corresponds to the one selected. In all cases the reference tissue was “lignotuber,” the reference period was “late winter,” and the reference treatment was “control.” Abbreviations: +, positive effect; −, negative effect; n.s., not significant; n.i., not included in the model; BAI, basal area increment; BA, basal area*.

## Appendix

**Table A1:** **Summary of the model for midday water potentials**.

**Fixed effects**	**Estimate**	***SE***	***DF***	***P*-value**
Intercept	−1.633	0.146	55	^***^
*P. latifolia*	−0.067	0.206	55	n.s.
*Q. ilex*	−0.317	0.206	55	n.s.
Spring	−0.350	0.206	55	n.s.
Summer	−1.450	0.206	55	^***^
Winter	−0.733	0.206	55	^***^
*P. latifolia*:Spring	0.100	0.292	55	n.s.
*Q. ilex*: Spring	0.483	0.292	55	n.s.
*P. latifolia*:Summer	−1.767	0.292	55	^***^
*Q. ilex*:Summer	0.167	0.292	55	n.s.
*P. latifolia*:Winter	0.767	0.292	55	^*^
*Q. ilex*:Winter	0.633	0.292	55	^*^

The reference specie was “A. unedo” and the reference season was “autumn.” The treatment effect was not included in the best model. Abbreviations:

* 0.01 < P < 0.05;

*** P < 0.001;

*ns, P > 0.05*.

**Table A2:** **Summary of the model for BAI growth**.

**Fixed effects**	**Estimate**	***SE***	***DF***	***P*-value**
Intercept	0.387	0.043	15231	^***^
*A. unedo*	0.797	0.075	1299	^***^
*P. latifolia*	−0.284	0.054	1299	^***^
Drought	−0.193	0.063	4	^*^
Period 2003–2012	0.080	0.026	15231	^**^
*A. unedo*: Drought	−0.609	0.120	1299	^***^
*P. latifolia*: Drought	0.209	0.078	1299	^**^
*A. unedo*: Period 2003–2012	−0.383	0.056	15321	^***^
*P. latifolia*: Period 2003–2012	−0.082	0.038	15321	^*^
Drought: Period 2003–2012	0.157	0.039	15321	^***^
*A. unedo*: Drought: Period 2003–2012	0.144	0.089	15321	n.s.
*P. latifolia*:Drought: Period 2003–2012	−0.141	0.054	15321	^**^

The reference specie was “Q. ilex,” the reference period was “1999–2002,” and the reference treatment was “control.” Abbreviations:

* 0.01 < P < 0.05;

** 0.001 < P < 0.01;

*** P < 0.001;

*ns, P > 0.05*.
